# Query-constraint-based mining of association rules for exploratory analysis of clinical datasets in the National Sleep Research Resource

**DOI:** 10.1186/s12911-018-0633-7

**Published:** 2018-07-23

**Authors:** Rashmie Abeysinghe, Licong Cui

**Affiliations:** 1 Department of Computer Science, University of Kentucky, Lexington, KY USA; 2 Institute for Biomedical Informatics, University of Kentucky, Lexington, KY USA

**Keywords:** Query-constraint-based association rule mining, National sleep research resource, Exploratory data analysis

## Abstract

**Background:**

Association Rule Mining (ARM) has been widely used by biomedical researchers to perform exploratory data analysis and uncover potential relationships among variables in biomedical datasets. However, when biomedical datasets are high-dimensional, performing ARM on such datasets will yield a large number of rules, many of which may be uninteresting. Especially for imbalanced datasets, performing ARM directly would result in uninteresting rules that are dominated by certain variables that capture general characteristics.

**Methods:**

We introduce a query-constraint-based ARM (QARM) approach for exploratory analysis of multiple, diverse clinical datasets in the National Sleep Research Resource (NSRR). QARM enables rule mining on a subset of data items satisfying a query constraint. We first perform a series of data-preprocessing steps including variable selection, merging semantically similar variables, combining multiple-visit data, and data transformation. We use Top-k Non-Redundant (TNR) ARM algorithm to generate association rules. Then we remove general and subsumed rules so that unique and non-redundant rules are resulted for a particular query constraint.

**Results:**

Applying QARM on five datasets from NSRR obtained a total of 2517 association rules with a minimum confidence of 60% (using top 100 rules for each query constraint). The results show that merging similar variables could avoid uninteresting rules. Also, removing general and subsumed rules resulted in a more concise and interesting set of rules.

**Conclusions:**

QARM shows the potential to support exploratory analysis of large biomedical datasets. It is also shown as a useful method to reduce the number of uninteresting association rules generated from imbalanced datasets. A preliminary literature-based analysis showed that some association rules have supporting evidence from biomedical literature, while others without literature-based evidence may serve as the candidates for new hypotheses to explore and investigate. Together with literature-based evidence, the association rules mined over the NSRR clinical datasets may be used to support clinical decisions for sleep-related problems.

**Electronic supplementary material:**

The online version of this article (10.1186/s12911-018-0633-7) contains supplementary material, which is available to authorized users.

## Background

Biomedical and clinical data has been generated at an unprecedented speed and scale [1, 2], providing researchers with significant opportunities for data-driven knowledge discovery in biomedicine [3]. The National Sleep Research Resource (NSRR) is one of such data repositories freely available to the sleep research community [4]. It aggregates and shares sleep-related clinical data as well as physiological signals generated from clinical trials and epidemiological cohort studies funded by the U.S. National Institutes of Health. Proper use of repositories like NSRR could aid in informed decision making and improve patient safety [2]. From a research perspective, they could be used in knowledge discovery to facilitate rapid generation or testing of hypotheses.

Association Rule Mining (ARM) is an exploratory data mining technique that has shown great potential in the biomedical domain for knowledge discovery. It is used extensively to find associations among variables that satisfy some predefined interestingness parameters. A potential issue of ARM, especially when directly used in large biomedical datasets, is that it will result in many uninteresting rules. For instance, demographic features of patients (e.g., gender and race) always appear in biomedical datasets, which may result in an overwhelming number of gender-related association rules with high support and confidence, which are dominant but less interesting. Another potential challenge of performing ARM in biomedical datasets is the existence of semantically similar variables. Rules containing such similar variables are of less interest because these variables capture similar or same characteristics. Therefore, it is often needed to apply certain techniques which address these issues and filter out those uninteresting rules.

In this paper, we introduce QARM, a query-constraint-based ARM method where the rules mined are based on a subset of data satisfying a certain query constraint. For example, if the criteria is “patients who have had a stroke”, then the generation of association rules will be only based on the subset of patients who have had a stroke, thus the rules obtained will be more relevant to the criteria of interest. Such query-constraint-based ARM empowers biomedical researchers to perform exploratory data analysis in large biomedical data repositories and generate or test potential hypotheses.

### National Sleep Research Resource (NSRR)

Launched in 2014, NSRR provides free access in a web-based portal to large collections of de-identified physiological signals and clinical data elements (or variables) collected in well-characterized cohorts and clinical trials to support research on risk factors and outcomes of sleep disorders [5]. Each de-identified patient record of NSRR contains clinical data elements including demographic information (e.g., age, gender, race), anthropometric parameters (e.g., height, weight), physiologic measurements (e.g., heart rate), medical history (e.g., asthma, cancer, diabetes, stroke), medications (e.g., anti-coagulant, benzodiazepine), sleep symptoms (e.g., problems falling asleep), and other symptoms (e.g., chronic cough) [4].

For each dataset in NSRR, the clinical data as well as the data dictionary are stored in comma-separated values (CSV) files. Here the data dictionary contains the metadata of the clinical data (e.g., data type, value domains). Since the NSRR datasets are collected from different sleep-related studies, there are both common and disparate data elements across diverse datasets. The common data elements are maintained in a Canonical Data Dictionary (CDD), and mappings are provided between the CDD elements and the data elements in each individual dataset. We refer to common data elements in the CDD as *canonical variables* and data elements in each individual dataset as *dataset variables*, respectively.

In this work, we use five datasets from NSRR: Cleveland Family Study (CFS), Childhood Adenotonsillectomy Trial (CHAT), Hispanic Community Health Study/Study of Latinos (HCHS/SOL), Heart Biomarker Evaluation in Apnea Treatment (HeartBEAT), and Sleep Heart Health Study (SHHS). The five datasets were chosen based on the availability of sufficient number of dataset variables mapping to canonical variables. More details about these datasets can be found in Table 1.

**Table 1:** Five NSRR datasets used in this work: CFS, CHAT, HCHS/SOL, HeartBEAT, and SHHS

Dataset	Number of	Age of	Timeframe of
	subjects	subjects	data collection
CFS	735	6-88	2001-2006
CHAT	1243	5-9	2007-2012
HCHS/SOL	16,415	18-76	2009-2013
HeartBEAT	318	45-75	2010-2012
SHHS	5804	40-89	1995-2010

Dataset variables in NSRR are typically imbalanced [6]. For example, the variable *stroke15* (MD Reported Stroke) in the SHHS dataset has two possible values: “yes” and “no”, with a distribution of 3.3 and 96.7% respectively (i.e., an imbalance rate [6] of 3.3%). In the SHHS dataset, the average imbalance rate of variables with yes/no values is 5.16% (see Table 2).

**Table 2:** The number of canonical variables used in each dataset, number of dataset variables to which the canonical variables map, average imbalance rate of dataset variables, and number of association rules obtained

Dataset	No. of	No. of mapped	Average	No. of
	canonical variables	dataset variables	imbalance rate	association rules
CFS	40	113	10.72%	898
CHAT	5	20	8.27%	29
HCHS/SOL	31	75	6.17%	661
HeartBEAT	13	31	12.60%	128
SHHS	50	138	5.16%	801

### Association Rule Mining (ARM)

Association rules can be formally defined as follows [3, 7–9]. Let *D*={*t*_1_,*t*_2_,....,*t*_*n*_} be a set of transactions and *I*={*i*_1_,*i*_2_,....,*i*_*m*_} be a set of items. Each transaction *t*_*i*_ in *D* contains a subset of the items in *I*, that is, *t*_*i*_⊂*I*. In association analysis, subsets of *I* are called itemsets. An association rule is defined as an implication of the form *X*→*Y*, where *X*,*Y*⊆*I* are two itemsets and *X*∩*Y*=*∅*. *X* and *Y* are called antecedent and consequent, respectively.

The strength of an association rule *X*→*Y* can be measured by *Support* (the proportion of transactions that contain both X and Y) and *Confidence* (the proportion of the transactions that contains *X* which also contains *Y*). Rules that satisfy the user-specified minimum support (*minsup*) and minimum confidence (*minconf*) thresholds are called strong association rules. They are the key elements obtained from an analysis of all possible rules [3].

There are various algorithms introduced for ARM [10, 11]. In this work we leverage the top-*k* non-redundant association rule mining algorithm [12].

### Top-*k* Non-Redundant (TNR) ARM Algorithm

Choosing suitable values for parameters *minsupp* and *minconf* may be done by trial which is time-consuming. In some cases, users may have limited resources to analyze the obtained rules and hence are only interested in finding a certain amount of rules (e.g. top-k rules). Fournier-viger et al. [12] introduced the top-*k* algorithm to address the problem of difficulty in selecting suitable values for parameters *minsupp* and *minconf*. In our query-constraint-based ARM, fine-tuning *minsup* and *minconf* parameters for each query constraint would be a difficult task, thus we choose top-k rules for exploratory analysis.

Fournier-viger et al. [13] later introduced the TNR algorithm to address the redundancy issues existing in the original top-*k* algorithm. The TNR algorithm takes *k* (the number of association rules to be found), *minconf* and *Δ* (exactness improving parameter) as parameters, and approximates top-*k* rules with the top support having a confidence above the *minconf* threshold. The algorithm shows good performance and scalability, and in situations where the user wants to control the number of rules obtained, it is an advantageous alternative to classical ARM algorithms.

### Related work

ARM has been widely used in biomedical domains to facilitate knowledge discovery and disease prediction. For example, Hu et al. [14] have introduced a semantic-based ARM method to discover hidden connections among biomedical concepts from disjoint biomedical literature sets. The discovered novel relations could be used by domain experts for purposes such as conducting new experiments, trying new treatments etc. Wang et al. [3] have described preliminary results of applying ARM techniques to University of Calgary Atlas of mammograms. They have proposed a new breast mass classification method based on quantitative ARM. Agrawal et al. [15] have done an ARM analysis on lung cancer data from the Surveillance, Epidemiology, and End Results (SEER) program to identify hotspots in the cancer data. These hotspots are where the patient survival time is significantly higher and lower than the average survival time. Ordonez et al. [9] have introduced an ARM method that uses search constraints to reduce the number of rules. It searches for association rules on a training set and then validates them on an independent test set. They have used this approach to predict heart diseases.

While ARM has been widely applied for knowledge discovery in biomedicine, query-constraint-based ARM which performs ARM on a subset of patients, has not been well investigated. This approach combines information retrieval with ARM, which would help biomedical researchers to perform exploratory analysis of datasets using query constraints.

## Methods

In this work, we introduce QARM, a query-constraint-based ARM method for exploratory analysis of biomedical datasets. First a series of data pre-processing steps are performed including variable selection, variable merging, combining multiple-visit data, and query-constraint-based data transformation. Then the top-k non-redundant ARM algorithm is used to mine association rules based on different query criteria on the five datasets in NSRR. Two post-processing steps are taken for removing general rules and subsumed rules.

### Variable selection

Each variable in NSRR datasets has a type (e.g., categorical, numerical). Each categorical variable has a domain defining the possible values of the variable. For example, in the SHHS dataset, *prev_hx_stroke (previous history of stroke)* is a categorical variable having a domain of which the possible values consist of “yes” and “no”; and the categorical variable *fstk_type (type of fatal stroke)* has a domain with possible values “hemorrhagic”, “intracerebral-hemorrhage”, “ischemic”, “isch-unknown”, “subarachnoid hemorrhage”, and “unknown”.

In this work, we mainly focus on categorial variables with domains of the yes/no type for simplicity. In addition, we choose variables with regard to patients’ medical history, medications, sleep symptoms, and other symptoms.

Based on the above variable selection criteria, we obtained a set of variables from the Canonical Data Dictionary (called *canonical variables*), as well as the study-specific variables which are mapped to the canonical variables for each individual dataset (called *dataset variables*). It is worth noting that one canonical variable may map to multiple dataset variables. Take the canonical variable *“strokehist (stroke - history)”* as an example. It maps to two dataset variables in the SHHS dataset: *“stroke15 (MD reported stroke)”* and *“prev_hx_stroke (previous history of stroke)”*; it maps to one dataset variable in the HeartBEAT dataset: *“dxstroke (diagnosed: stroke)"*; and it maps to one dataset variable in the CFS dataset: *“strodiag (physician-diagnosed stroke)"*. In addition, a query constraint can be any canonical variable with value “yes". For instance, *“strokehist (stroke - history)”* with value “yes" can serve as a query constraint.

### Variable merging

Since certain variables in a dataset may capture similar information, association rules obtained including such similar variables would be of less interest. For example, both variables *prev_hx_stroke (previous history of stroke)* and *stroke15 (MD reported stroke)* in SHHS capture the information about whether a patient has had a stroke. Occurences of such variables together in a rule might make it uninteresting, e.g., *{prev_hx_stroke}* →*{stroke15}*.

Therefore, we merge such variables before performing QARM to avoid obtain association rules with such similar variables. This is done such that whenever a patient exhibits a “yes” to at least one of the similar variables, then the value of the merged variable will also be “yes”. Here, the dataset variables mapping to the same canonical variable are considered similar, and hence merged. We refer to this method as the “merged method”. For comparison, we also performed QARM without such a merging, which we refer to as “unmerged method”. The latter is only used for the purpose of comparison with the “merged method”. Therefore, unless otherwise specifically mentioned, in all the scenarios we are using the “merged method”.

### Combining multiple-visit data

In NSRR, some datasets contain patient data collected in multiple visits. For instance, the datasets CHAT, HeartBEAT and SHHS contain data collected in two patient visits. These multiple visits of a dataset were combined into one as a preprocessing step before QARM was performed. Since multiple visits may contain data collected for the same variable, the combination was performed as follows: for the same patient, if the value of the variable appear as “yes” in at least one of the visits, then the combined result will be “yes”; otherwise, the combined result will be “no”. For example, in the CHAT dataset, the variable “*med1c1 (ever had asthma?)*” appears in both the baseline visit and follow-up visit; for the same patient, the combined result is “yes” as long as one of the visits has the “yes” value.

### Query-constraint-based data transformation

Given a query constraint, the clinical data of patients satisfying the query criteria needs to be transformed to a suitable format before being fed into the TNR algorithm. In clinical datasets like NSRR, the possible values of a patient variable with the domain of yes/no type may be “yes", “no", or “unknown" (or “NA"). This way it is clear whether the patient has the characteristic specified in the variable (“yes"), or the patient does not have the characteristic (“no"), or the information is unknown or not available. While “no" and “unknown" are important for capturing more precise information of patients, they may not be useful for generating association rules. For example, most patients in the SHHS dataset have not had a stroke (i.e., *stroke15* =*“no"* and *prev_hx_stroke* =*“no"*), in which cases the variables are imbalanced towards “no" values. If the “no" values for such variables were used for generating association rules (denoting the characteristics patients do not have), then it would have produced a lot of uninteresting and irrelevant rules also making the ARM process slow. Therefore, in this work, we only consider the “yes" values of variables for patient records satisfying the query criteria.

### QARM using TNR algorithm

Given a query constraint, QARM using TNR algorithm was applied to the patient data satisfying the query constraint after data transformation, with *k*=100, *minconf*=60*%* and *Δ*=10. For example, if the query constraint is the canonical variable *strokehist (stroke history)* based on the SHHS dataset, then only patients with *stroke15 (MD reported stroke)* =*“yes"* or patients with *prev_hx_stroke (previous history of stroke) = “yes"* will be selected for QARM, since the canonical variable *strokehist* maps to two dataset variables *stroke15* and *prev_hx_stroke*. This is as if selecting a sub-dataset with patients who have had a stroke and then performing QARM on it. We set a lower-bound of 20 to the number of patient records exhibiting this query constraint characteristic as a condition for the applicability of QARM so that a sufficient number of patient records will be considered. Here, we used the implementation of TNR in the SPMF open-source data mining library [16]. After QARM is performed, we sort the obtained association rules first by their *support* and then by their *confidence*.

Note that the *support* and the *confidence* of the obtained rules are based on the sub-dataset of patients satisfying the query constraint, not the entire dataset. In addition, the query constraint itself is not included to perform QARM since it is satisfied by each patient record in the sub-dataset.

### Removing general rules

For a given query constraint, the resulting rule set may contain rules which are generally observed throughout the whole dataset. In other words, such rules are not unique to patients exhibiting the query constraint characteristic, but general to majority of the patients in the dataset. Therefore, we eliminate such rules as follows. Assume that *O* is the set of top-*k* rules obtained for patients satisfying the query constraint. We further apply the TNR algorithm to obtain another set *N* of top-*k* rules for those patients who do not satisfy the query constraint. Then we remove the common rules (*O*∩*N*) from *O*, i.e., *O*−(*O*∩*N*) or *O*−*N*.

### Removing subsumed rules

The TNR algorithm defines redundancy in terms of Minimum Condition Maximum Consequent Rules as follows [13]. An association rule *r*_*a*_:*X*→*Y* is *redundant* with respect to another rule *r*_*b*_:*X*_1_→*Y*_1_ if and only if:

1. *confidence*(*r*_*a*_)=*confidence*(*r*_*b*_) and *support*(*r*_*a*_)=*support*(*r*_*b*_); and
2. *X*_1_⊆*X* and *Y*⊆*Y*_1_.

Satisfaction of both conditions is important in determining redundant rules during the ARM process. However, the resulting rule set may contain rules which satisfy condition 2 but not condition 1. Exploring such subsumed rules may not help the user in determining interesting associations among patient characteristics. Therefore, as a post-processing step, we remove all such rules which are subsumed by another rule. Note that removing common and subsumed rules may lead to a less number of rules (≤*k*) in the result.

## Results

A total of 71 canonical variables were obtained after the variable selection process. Since each canonical variable can serve as a query constraint, we interchangeably use terms “canonical variable" and “query constraint" in the followings. Table 2 shows the numbers of canonical variables identified in each of the five datasets, the numbers of mapped dataset variables corresponding to the canonical variables, and the numbers of association rules obtained within each dataset. It can be seen that SHHS covered the most number of canonical variables. In Table 2, a canonical variable used in an individual dataset is based on the existence of mapped dataset variables, as well as the existence of a considerable number of patients exhibiting the characteristic specified in the variable (at least 20 patients).

### Summary results

A total of 2517 association rules were obtained by applying QARM within each of the five datasets, using top *k* = 100 rules with a *minconf* threshold of 60% and *Δ* = 10. On average a query resulted in 18 rules.

Table 3 contains the resulting association rules obtained for the query constraint *strokehist (stroke-history)* in the SHHS dataset. For example, {*myocardial infarction-history*} → {*hypertension-history*} is an obtained association rule for the query. This indicates that for a patient who have had a stroke, if the patient happens to have myocardial infarction, they are likely to have hypertension as well.

**Table 3:** Resultant association rules for the query constraint “*strokehist (Stroke-history)*” in SHHS dataset

Antecedent	Consequent
Habitual snoring	Nonsteroidal anti-inflammatory drug, hypertension-history
Nonsteroidal anti-inflammatory drug	Habitual snoring, hypertension-history
Hypercholesterolemia	Hypertension-history, hmg-coa reductase inhibitor
Chronic obstructive pulmonary disease/emphysema-history	Nonsteroidal anti-inflammatory drug, hypertension-history
Chronic obstructive pulmonary disease/emphysema-history	Habitual snoring, hypertension-history
Loop diuretic	Hypertension-history
Hypercholesterolemia	Habitual snoring, hmg-coa reductase inhibitor
Myocardial infarction-history	Hypertension-history
Angina pectoris	Nonsteroidal anti-inflammatory drug, hypertension-history

### Merged method versus unmerged method

**Fig. 1:**
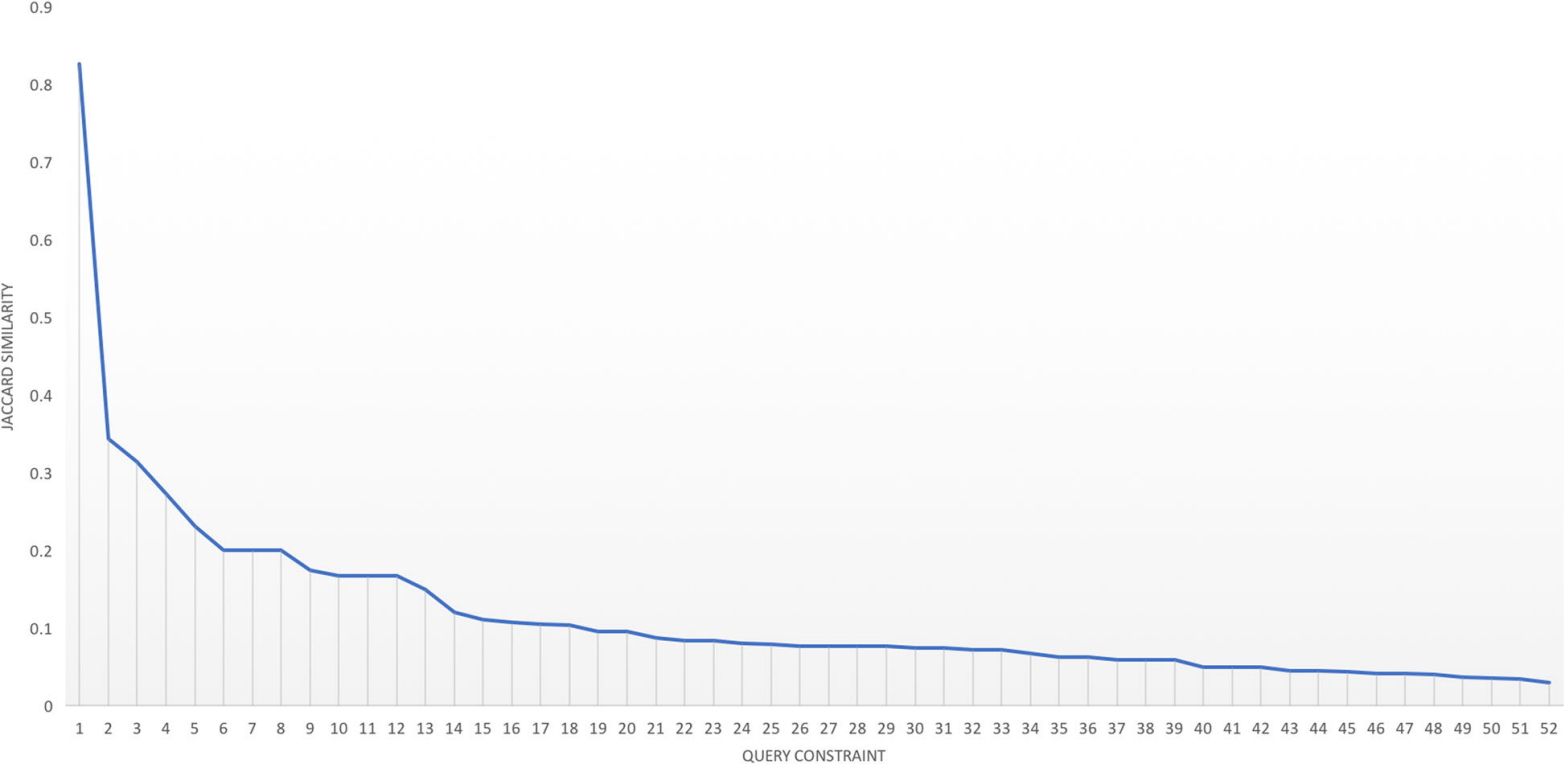
Jaccard similarity of queries having common rules by merged and unmerged methods

We also performed QARM using the “unmerged method” for comparison with the “merged method". Table 4 shows the numbers of common and distinct rules obtained by the “merged” and “unmerged” methods for 10 query constraints. For example, the query constraint *htnhist (hypertension-history)* derived 19 common rules by both the “merged” and “unmerged” methods, 1 distinct rule that is uniquely obtained by the “merged method”, and 3 distinct rules that are uniquely obtained by the “unmerged method”. Figure 1 contains a plot of Jaccard similarity values for result sets of merged and unmerged methods for the 52 queries where common rules were found between merged and unmerged methods. The first 10 queries in Fig. 1 refer to the 10 queries in Table 4.

**Table 4:** Numbers of common and distinct rules obtained by merged and unmerged methods for 10 query constraints

Description	Variable	Dataset	No. of	No. of distinct	No. of distinct
			common rules	rules (merged)	rules (unmerged)
Hypertension-history	htnhist	HeartBEAT	19	1	3
Thiazolidinedione	tzd	SHHS	11	10	11
Biguanide	biguanide	SHHS	11	15	9
Congestive heart failure-history	chfhist	SHHS	9	15	9
Typical Antipsychotic	typicalantipsychot	HCHS	6	20	0
Angiotensin 2 receptor blocker	arb	SHHS	3	10	2
Coronary artery disease-history	cadhist	HCHS	4	15	1
Potassium salt	potassiumsalt	SHHS	2	7	1
Cardiovascular disease-history	cvdishist	SHHS	4	9	10
Pacemaker placement	ppmhist	SHHS	6	19	11

### General and subsumed rules removed

Table 5 contains the number of general and subsumed rules removed for 10 query constraints. On average 36 general rules and 42 subsumed rules are removed from resultant rules of a query constraint.

**Table 5:** Numbers of general and subsumed rules removed

Description	Variable	Dataset	No. of	No. of general	No. of subsumed
			rules obtained	rules removed	rules removed
Depression	depresshist	HeartBEAT	2	62	36
Diabetes mellitus-history	dmhist	HeartBEAT	2	74	24
Myocardial infarction-history	mihist	HeartBEAT	4	70	26
l-triiodothyronine	triiodothy	SHHS	7	89	4
Chronic obstructive pulmonary	copdhist	HeartBEAT	7	68	25
Disease/emphysema-history					
Histamine-2 receptor Antagonist	h2blocker	SHHS	7	86	7
Anxiety disorder	anixietyhist	HeartBEAT	7	55	38
Asthma	asthmahist	HeartBEAT	7	64	29
Nonsteroidal Anti-inflammatory	nsaid	HCHS	8	86	6
Drug					
Stroke-history	strokehist	SHHS	9	80	11

## Discussion

In this work, we investigated a query-constraint-based ARM method which we applied to five clinical datasets in NSRR. We also investigated the common and distinct association rules obtained using the merged method versus unmerged method.

### Literature-based evidence to obtained association rules

Data mining techniques have been previously employed in clinical decision support systems for diagnosis, prediction and treatment of diseases [17, 18]. The association rules obtained based on the clinical datasets in NSRR may provide evidence for making clinical decisions for sleep-related problems together with further literature-based evidence.

Table 6 contains some preliminary findings of the supporting evidence from biomedical literature for 20 randomly chosen rules for the queries found in Table 4. For each query constraint, two rules have been randomly chosen.

**Table 6:** Randomly chosen example association rules obtained for queries in Tables 4 and 5 and supporting literature

Variable/Dataset	Description	Antecedent	Consequent	Supporting
				Literature
htnhist/HeartBEAT	hypertension-history	diabetes mellitus-history	hypercholesterolemia-history	None
htnhist/HeartBEAT	hypertension-history	diabetes mellitus-history	habitual snoring	[22–24]
tzd/SHHS	thiazolidinedione	hypertension-history	sulfonylurea, diabetes mellitus-history	None
tzd/SHHS	thiazolidinedione	hmg-coa reductase inhibitor	sulfonylurea, habitual snoring,	None
			nonsteroidal anti-inflammatory drug,	
			hypercholesterolemia	
biguanide/SHHS	biguanide	hypercholesterolemia	sulfonylurea, hypertension-history,	None
			hmg-coa reductase inhibitor	
biguanide/SHHS	biguanide	hypertension-history	sulfonylurea, hmg-coa reductase inhibitor,	None
			hypercholesterolemia	
chfhist/SHHS	congestive heart failure-history	angina pectoris	myocardial infarction-history	[32, 33]
chfhist/SHHS	congestive heart failure-history	loop diuretic	hypertension-history,	[19–21]
			angiotensin converting enzyme inhibitor	
typicalantipsychot/HCHS	Typical Antipsychotic	coronary artery disease-history	tricyclic antidepressant	None
typicalantipsychot/HCHS	Typical Antipsychotic	diabetes mellitus-history	tricyclic antidepressant	[34–36]
arb/SHHS	angiotensin 2 receptor blocker	chronic obstructive pulmonary	nonsteroidal anti-inflammatory drug,	[37–40]
		disease/emphysema-history	habitual snoring, hypertension-history	
arb/SHHS	angiotensin 2 receptor blocker	hypertension-history	nonsteroidal anti-inflammatory drug,	
			habitual snoring	None
cadhist/HCHS	coronary artery disease-history	angiotensin converting	thiazide diuretic, hypertension-history,	[22, 25, 26]
		enzyme inhibitor	diabetes mellitus-history	
cadhist/HCHS	coronary artery disease-history	persistent wheezing	hypertension-history	None
potassiumsalt/SHHS	potassium salt	loop diuretic	habitual snoring, hypertension-history	None
potassiumsalt/SHHS	potassium salt	habitual snoring	nonsteroidal anti-inflammatory drug,	
			hypertension-history	None
cvdishist/SHHS	cardiovascular disease-history	angina pectoris	nonsteroidal anti-inflammatory drug,	None
			habitual snoring, hypertension-history	
cvdishist/SHHS	cardiovascular disease-history	myocardial infarction-history	hypertension-history	[41]
ppmhist/SHHS	pacemaker placement	l-triiodothyronine	nonsteroidal anti-inflammatory drug	None
ppmhist/SHHS	pacemaker placement	nonsteroidal	chronic obstructive pulmonary	None
		anti-inflammatory drug	disease/emphysema-history,	
			hypertension-history, habitual snoring	
depresshist/HeartBEAT	depression	chronic obstructive pulmonary	hypercholesterolemia-history,	None
		disease/emphysema - history	habitual snoring, hypertension-history	
depresshist/HeartBEAT	depression	anxiety disorder	hypercholesterolemia-history,	None
			habitual snoring, hypertension-history	
dmhist/HeartBEAT	diabetes mellitus-history	anxiety disorder	depression, habitual snoring	[42–45]
			hypertension-history	
dmhist/HeartBEAT	diabetes mellitus-history	asthma	hypercholesterolemia-history,	[40, 46–48]
			habitual snoring, hypertension-history	
mihist/HeartBEAT	myocardial infarction-history	chronic obstructive pulmonary	hypercholesterolemia-history,	None
		disease/emphysema-history	habitual snoring, hypertension-history	
mihist/HeartBEAT	myocardial infarction-history	depression,	hypercholesterolemia-history,	None
		diabetes mellitus-history	hypertension-history	
triiodothy/SHHS	I-triiodothyronine	hypercholesterolemia	habitual snoring,	None
			hmg-coa reductase inhibitor	
triiodothy/SHHS	I-triiodothyronine	hypercholesterolemia	nonsteroidal anti-inflammatory drug,	None
			hmg-coa reductase inhibitor	
copdhist/HeartBEAT	chronic obstructive pulmonary	anxiety disorder	hypercholesterolemia-history,	[48–51]
	disease/emphysema-history		habitual snoring, hypertension-history	
copdhist/HeartBEAT	chronic obstructive pulmonary	asthma	hypercholesterolemia-history,	None
	disease/emphysema-history		habitual snoring	
h2blocker/SHHS	histamine-2 receptor antagonist	angiotensin converting	nonsteroidal anti-inflammatory drug,	None
		enzyme inhibitor	habitual-snoring, hypertension-history	
h2blocker/SHHS	histamine-2 receptor antagonist	hypertension-history	nonsteroidal anti-inflammatory drug,	None
			habitual snoring	
anixietyhist/HeartBEAT	anxiety disorder	habitual snoring	hypercholesterolemia-history,	[40, 44, 48, 52]
			depression, hypertension-history	
anixietyhist/HeartBEAT	anxiety disorder	myocardial infarction-history	hypercholesterolemia-history,	[48, 53, 54]
			depression, hypertension-history	[44]
asthmahist/HeartBEAT	asthma	depression	chronic obstructive pulmonary	None
			disease/emphysema-history,	
			hypercholesterolemia-history	
asthmahist/HeartBEAT	asthma	hypertension-history	diabetes mellitus-history	[55, 56]
nsaid/HCHS	nonsteroidal	hypertension-history	thiazide diuretic	[57, 58]
	anti-inflamatory drug			
nsaid/HCHS	nonsteroidal	leukotriene receptor	asthma, persistent wheezing	None
	anti-inflamatory drug	antagonist		
strokehist/SHHS	stroke-history	myocardial infarction-history	hypertension-history	[41, 59]
strokehist/SHHS	stroke-history	chronic obstructive pulmonary	nonsteroidal anti-inflammatory drug,	None
		disease/emphysema-history	hypertension-history	

For example, consider the rule {*loop diuretic*} → {*hypertension-history, angiotensin converting enzyme inhibitor*} for the query constraint *congestive heart failure-history* in SHHS dataset. According to [19], a combined treatment with low doses of *loop diuretics* and *angiotensin converting enzyme inhibitors* can be used to treat *hypertension* without adverse reactions associated with larger doses of either therapy alone. *Loop diuretics* and *angiotensin converting enzyme inhibitors* alone are used to treat *hypertension*. So, these facts support this rule which states, whenever a patient is using *loop diuretics*, he or she is more likely to have *hypertension* and be treated with *angiotensin converting enzyme inhibitor*. The existence of this rule among patients with *congestive heart failure* can be validated by [20, 21], which states *loop duretics* are widely used to treat *congestive heart failure*.

Araki et al. [22] mention that *hypertension* is a common *diabetes* comorbidity. According to [23, 24] there exists an association between *habitual snoring* and *diabetes mellitus* prominently in women. Therefore, these facts found in literature supports the rule {*diabetes mellitus-history*} → {*habitual snoring*} for the query constraint *hypertension-history* in HeartBEAT dataset.

Consider the rule *angiotensin converting enzyme inhibitor*} → {*thiazide diuretic, hypertension-history, diabetes mellitus-history*} for query constraint *coronary artery disease-history* in HCHS dataset. According to [25], *angiotensin-converting enzyme inhibitors* are both used to treat *hypertension* and *coronary artery disease*. Chowdhury et al. [26] state that both *angiotensin-converting enzyme inhibitors* and *thiazide diuretics* are used for the treatment of *hypertension*. As mentioned earlier, *hypertension* is a common *diabetes* comorbidity [22]. So these facts found in literature supports the above mentioned rule.

According to [27], *sulfonylureas* are oral antidiabetic agents. However, they may cause *hypertension* by their extra-pancreatic effects [28]. Sehra et al. also mention that within a few years of diagnosis, patients with *type 2 diabetes mellitus* develop *hypertension*. Therefore, the rule {*hypertension-history*} → {*sulfonylurea, diabetes mellitus-history*} which states that whenever a patient is having *hypertension*, he or she is more likely to be using *sulfonylurea* and having *diabetes-mellitus* is supported by the given evidence. However, we could not find any evidence that this rule is specific to patients using *thiazolidinedione*. So, this seems like a general rule which has not been removed during the general rule removal. A larger *k* value may have removed this from the result set.

For those rules with no supporting evidence found in literature, they may serve as candidates for generating new hypotheses for further discovery and investigation.

### Distinction with related work

ARM has been widely applied to biomedical datasets for data-driven knowledge discovery. However, exploratory ARM based on a particular query constraint has been rarely investigated. QARM would allow researchers to perform exploratory analysis based on a subset of data of interest by composing a specific query criteria to filter out irrelevant data.

The heuristic of our approach is to some extent similar to that of traditional constraint-based mining [29], which enables users to specify constraints to confine the search space. In another related work, Kubat et al. [30] have presented an approach that converts a market-based database into an itemset tree to get a quick response to targeted association queries. Our approach differs from other constraint-based mining approaches [29] and targeted association querying [30], in that we directly apply the query constraint on the input data before starting the mining process rather than applying it to the output rules or applying it during the mining process. Another important distinction is that unlike other approaches that always include the constraint in the mined rules, the rules mined by our approach do not contain the query constraint itself. Although one of the motivations behind QARM is to reduce the number of uninteresting rules generated from an imbalanced dataset, it is not used to address the issue of the imbalance of the dataset. To the best of our knowledge, constraint-based mining has not been employed for the reducing purpose before. Furthermore, in terms of the datasets used, this is the first rule-mining-based work on analyzing NSRR datasets.

We performed a preliminary study [31] on query-constraint-based ARM in NSRR which motivated this work. However, in [31] we did not perform any post-processing on the results. The results contained a lot of general as well as subsumed rules. To address this issue, in this work, we have introduced two post-processing steps to remove such rules from the results so that a concise, interesting rule set will be provided as the output for a query. From Table 5 it could be noted that a large potion of rules were removed as a result of these two steps. In addition, we also perform a literature survey to validate a random sample of the rules obtained.

### Merged versus unmerged

It was noted that some of the rules obtained distinctly by the unmerged method are not interesting, since they contain rules which have similar dataset variables. For example, for the query constraint *thiazolidinedione* in SHHS, there exists a rule in the form of {*sulfonylurea*} → {*sulfonylurea, hypertension-history*} which is not interesting due to the existence of the similar variable *sulfonylurea* in multiple locations of the rule. Therefore, merging similar variables serves as a means of filtering such uninteresting rules.

From Fig. 1, it could be noted that for most queries, the resultant rules of merged and unmerged methods are quite different. Although it was observed that unmerged method obtains uninteresting rules with similar variables while the merged method does not, further analysis is needed to confirm what factors contributed to this difference.

It was also noted that the unmerged method obtains a significantly lower number of association rules than the merged method. Using *k* = 100, the unmerged method obtained 653 rules in total across all the datasets for all query constraints while the merged method obtained a total of 2517. This is because the unmerged method obtained a lot of subsumed rules in the following format. Consider the rules {*hypertension (shhs2)*} → {*sleep habits (shhs1): ever snored*} and {*self-reported hypertension (shhs1)*} → {*sleep habits (shhs1): ever snored*} obtained for the query constraint *stroke-history* in SHHS dataset using the unmerged method. Both these rules contains similar variables *hypertension (shhs2)* and *self-reported hypertension (shhs1)* as antecedents and the same variable *sleep habits (shhs1): ever snored* as the consequent. Therefore, these rules actually could be considered similar because they convey the same association: {*hypertension-history*} → {*habitual snoring*}. Unmerged method produced a large number of such rules which were filtered during the subsumed rule removal.

### Limitations and future work

In this work, we only considered categorical variables with domains of the *yes/no* type for the query-constraint-based ARM. Other categorical variables involve complex domains which need to be manually examined to determine whether they are meaningful for rule mining, and thus we expect to explore them in future work. It would also be interesting to further investigate numerical variables, where numerical values can be categorized into some predefined ranges. In addition, we only considered query constraints involving a single canonical variable, however, it can be generalized to query constraints consisting of multiple canonical variables.

In the future we would like to perform an automated literature-based analysis as well as a manual review by clinical experts to validate the obtained rules. We also plan to incorporate QARM in a web-based system for biomedical researchers to dynamically compose query constraints and interactively perform exploratory data analysis in NSRR. We used top 100 rules when performing QARM in this paper. To support interactive exploratory analysis, such parameters could be configured and decided by the end users.

## Conclusion

In this paper, we applied QARM, a query-constraint-based association rule mining method, to five diverse clinical datasets in the National Sleep Resource Resource. QARM shows the potential to support exploratory analysis of large biomedical datasets by mining a subset of data satisfying a query constraint. It is also shown as a useful method to reduce the number of uninteresting association rules generated from imbalanced datasets. Our analysis indicates that merging similar variables in datasets is an effective method to filter uninteresting rules. Also, removing general and subsumed rules resulted in more concise and interesting rules. A preliminary literature-based analysis showed that some association rules have supporting evidence from biomedical literature, while others without literature-based evidence may serve as the candidates for new hypotheses to explore and investigate.

## Additional file

Additional file 1 Results obtained: Results.zip contains the results obtained by merged and unmerged methods for different query constraints across the five datasets in NSRR. (ZIP 199 kb)

## Abbreviations

ARM: Association rule mining
QARM: Query-constraint-based ARM
